# Correlation of Copper and Zinc in Spontaneous Abortion

**DOI:** 10.22074/ijfs.2019.5586

**Published:** 2019-04-27

**Authors:** Riddhi Thaker, Hina Oza, Idrish Shaikh, Sunil Kumar

**Affiliations:** 1 Division of Reproductive and Cytotoxicology, ICMR-National Institute of Occupational Health, Ahmedabad, India; 2 Department of Obstetrics and Gynecology, Civil Hospital, Ahmedabad, India

**Keywords:** Copper, Pregnancy Outcome, Spontaneous Abortion, Trace Elements, Zinc

## Abstract

**Background:**

Humans require minute amounts of trace metals to maintain body’s normal growth and physiological functions; such elements may also play a vital role in pregnancy and pregnancy outcome. The present study was con- ducted to assess the role of two trace metals, zinc (Zn) and copper (Cu) in women with history of spontaneous abortion (SAb cases) in comparison to women without such history (controls).

**Materials and Methods:**

In this retrospective study, a total of 277 subjects were enrolled from the Obstetrics and Gyne- cology Department, Civil Hospital, Ahmedabad, India. Personal demographic information, medical history, reproductive history especially details of number of SAb, duration of last SAb, number of children, etc. were recorded using pre- designed and pre-tested proforma. Serum Zn and Cu levels were measured by an atomic absorption spectrophotometer.

**Results:**

The data indicated that the serum level of Cu (P<0.01) and Zn was lower in SAb cases as compared to controls. Correlation between the number of SAbs and trace metals levels showed a significant negative correlation between Cu and Cu/Zn and the number of SAbs. Cu/Zn was higher in controls and women having at least one child as compared cases and women without child, respectively. Pregnant women had higher levels of trace elements as compared to non-pregnant women at the time of enrollment.

**Conclusion:**

The data revealed that trace metals such as Zn and Cu have a positive role in pregnancy outcome and optimum levels of Zn and Cu might be able to decline the chances of SAb occurrence in addition to other factors. The ratio of Cu/Zn has a positive role in reproductive outcomes.

## Introduction

In the advent of industrialization and increasing need for foodstuffs and other requirements for ever growing population, the levels of toxic substances in the environment have increased considerably. Some heavy metals are toxic to humans especially to the pregnant women and developing fetus, even at very low doses, also. However, some trace metals are necessary for normal growth, development and various other physiological functions. Trace elements include more than 60 substances that are generally present at low concentrations in the environment and mammalian tissues. They are present in tissues and serum at a very low concentration (i.e. within picogram or microgram levels), and their absorption, distribution, storage and excretion are firmly controlled. At least a dozen of trace elements are considered essential minerals for human (1). Earlier, it is reported that trace metals like zinc (Zn), copper (Cu), selenium, chromium, cobalt, iodine, manganese, and molybdenum are indispensable for human body and these trace elements accounts for only 0.02 % of the total human weight and play noteworthy role in physiological functions of the body (2). Their deficiencies can lead to reduced activities of associated enzymes and cellular function.

Recently Prashanth et al. (3) reported that trace elements facilitate various vital biochemical reactions by acting as cofactors for many enzymes, and stabilizing structures of enzymes and proteins and they are significant for cell function at biological, chemical and molecular levels. Some of the trace elements govern vital biological processes by binding molecules on the receptor site on cell membrane or by altering the structure of membrane to avert the entry of specific molecules into the cell. At optimum concentrations, they are significant for maintenance of cellular structures, but at insufficient levels, they may adopt different pathways and cause diseases. Earlier, Savory and Wills (4) reported that trace metals play a vital role in biological processes, either as indispensable components or toxins. Pathak and Kapil (5) also reported that inadequacies of trace metals such as Cu, Zn and magnesium were implicated in various adverse reproductive events such as infertility, congenital anomalies, pregnancy wastage, pregnancy-induced hypertension, premature rupture of membranes, placental abruption, still births, low birth weight, etc.

Several reports indicated that optimum concentrations of trace metals are also essential for favorable pregnancy and pregnancy outcome. One such pregnancy outcome focused here is spontaneous abortion (SAb). SAb, or miscarriage, is a clinically acknowledged pregnancy loss before the 20^th^ week of gestation (6, 7). The World Health Organization defines SAb as expulsion or withdrawal of an embryo or fetus weighing 500 g or less. Whereas recurrent pregnancy loss is generally defined as 3 consecutive pregnancy losses prior to 20 weeks from the last menstrual period (8). Ajayi et al. (9) reported that decline in vital micronutrients such as Zn, Cu and vitamin E may be associated with recurrent SAb. Both Zn and Cu are essentials to the body but Cu to Zn ratio is clinically more important as compared to the concentration of either elements alone (10). Recently Shen et al. (11) reported that trace elements are closely linked with fetal growth and development throughout pregnancy and their shortage can lead to adverse pregnancy outcomes. Jariwala et al. (12) found that Zn and selenium levels were lowered in pregnant mothers supporting the idea of the need of Zn and selenium supplementation along with iron during pregnancy. Thus, the present study was conducted to understand the role of Zn and Cu with respect to SAb.

## Materials and Methods

In this retrospective study, a total of 277 subjects (118 control-subjects without history of SAb and bearing at least one child, and 159 case-subjects with history of SAb) were enrolled from the Out-Patient Department (OPD), Obstetrics and Gynecology, Civil Hospital, Ahmedabad, India. The control subjects (n=118) included 74 pregnant women with children enrolled from OPD and 44 non-pregnant with children enrolled from ward. While SAb cases (n=159) included 86 pregnant women with history of SAb enrolled from OPD and 73 non-pregnant (at the time of enrolment) recruited from ward with history of SAb/current SAb faced. An informed consent was obtained from each participant after explaining the aims and objective of the study as well as benefit of the study in general to the society. The ethical approval of the study was attained from the Institutional Human Ethical Committee of National Institute of Occupational Health (NIOH), Ahmedabad. The personal demographic information, habits, medical history, reproductive history especially details of number of SAb, duration of last SAb, number of children born, etc. were collected and recorded on pre-designed and pre-tested proforma through questionnaire interviews.

Blood samples (about 2 ml) were collected from each subjects and serum was separated using centrifugation (REMI R-8C, India) at 3000 rpm and kept in different aliquots in deep freeze till analysis. The serum levels of Zn and Cu were measured at 213.9 and 324.8 nm, respectively using an atomic absorption spectrophotometer (model no: AAnalyst-800, Perkin Elmer, USA) after preparing proper dilutions. The data were computerized using Microsoft Excel and presented as mean ± SE. Independent student’s t test and one-way ANOVA were applied with a significance level of P<0.05 to analyze the data using SPSS 16 (SPSS Inc., Chicago, USA). Also, Cu/Zn ratio with respect to different categories/variables was also determined.

## Results

The characteristics of both SAb (cases) and control subjects, is depicted in Table 1. The mean age of the SAb group was more than a year higher than that of the control group. Most of the cases of both SAb and control groups were residing in residential area; however, almost about 15-16% of subjects were living in agricultural or industrial areas. Further, about 92 and 88% subjects were literate in control and SAb groups, respectively and about 22 and ~10 % of SAb and control subjects were employed, respectively. Further, about 75% of SAb subjects had a history of one SAb and 20 and 5% of subjects had a history of two and more than 2 SAb, respectively. Further, all the control subjects were having children while about 34.6% SAb group subjects did not have children and the rest of them had children and a history of SAb (Table 1).

**Table 1:** Characteristics of study population (control and SAb cases)


Characteristic		Cases n=159	Controlsn=118

Mean age at the time of SAb/child birth^*^		24.85 ± 0.32	23.65 ± 0.33
	Area of residence		
	Agricultural area	10 (6.29)	5 (4.24)
	Industrial area	16 (10.06)	13 (11.02)
	Residential area	133 (83.65)	100 (84.75)
Educational status			
	Illiterate	19 (11.95)	9 (7.63)
	Literate	140 (88.05)	109 (92.37)
Employment status			
	Employed	36 (22.64)	12 (10.17)
	Unemployed	123 (77.36)	106 (89.83)
Pregnancy status			
	Pregnant (at the time of interview and sample collection)	86 (54.09)	74 (62.71)
	Non-pregnant (at the time of interview and sample collection)	73 (45.91)	44 (37.29)
Number of SAb			
	One SAb	119 (74.84)	-
	Two SAb	32 (20.13)	-
	More than two SAb	8 (5.03)	-
Mean gestational age at the time of SAb (in weeks)		10.37 ± 0.32	-
Pregnancy loss in trimester			
	First trimester	117 (73.58)	
	Second trimester	27 (16.98)	
PL in 1^st^ or 2^nd^ trimester (subjects more than one SAb, one SAb in 1^st^ trimester and another SAb in 2^nd^ trimester)		15 (9.43)	
Number of children			
	0 child	55 (34.59)	-
	1 child	68 (42.77)	63 (53.39)
	2 children	23 (14.47)	41 (34.75)
	3-4 children	13 (8.18)	14 (11.86)


Data are mean ± SE or n (%). SAb; Spontaneous abortion, PL; Pregnancy loss, and *; Calculated as: age at interview-duration of last SAb (cases)/child birth (control).

The data revealed that the mean serum levels of Cu and Zn were higher in control subjects as compared to SAb subjects. There was a significant difference between case and control groups with respect to Cu levels. Further, it was observed that the levels of these trace metals in the pregnant women were higher than non-pregnant women at the time of enrollment. Serum Cu level was higher and serum Zn level was lower in women bearing child in comparison to women bearing no child; however, these differences were statistically non-significant (Table 2). Moreover, Cu/Zn ratio was higher statistically non-significant in controls compared to SAb cases.

**Table 2:** Level of Serum Zn and Cu with respect to reproductive history


Group	Serum Cu (mg/L)	Serum Zn (mg/L)	Cu/Zn

Case n=159	1.59 ± 0.05	1.430 ± 0.03	1.28 ± 0.066
Control n=118	1.81 ± 0.06^**^	1.463 ± 0.05	1.46 ± 0.091
P value	0.008	0.594	0.107
Pregnant n=160^#^	1.75 ± 0.05	1.491 ± 0.03	1.34 ± 0.067
Non-pregnant n=117^##^	1.59 ± 0.06	1.380 ± 0.04	1.38 ± 0.091
P value	0.103	0.071	0.728
With childn=222	1.714 ± 0.04	1.417 ± 0.03	1.40 ± 0.063
Without childn=55	1.584 ± 0.09	1.553 ± 0.06	1.17 ± 0.104
P value	0.231	0.075	0.061


The data are presented as mean ± SE. SAb; Spontaneous abortion, Cu; Copper, Zn; Zinc, **; P<0.01 show significant differences between the two groups based on independent student’s t test, ^#^; Pregnant 160 includes 74 pregnant women having previous child birth and 86 case women with history of SAb, and ^##^; Non-pregnant 117 includes 44 non-pregnant with previous child birth and 73 non-pregnant with present and past history of SAb, at the time of enrolment.

Besides, the levels of trace metals were analyzed with respect to lifestyle habits of the study population. The data revealed that women having vegetarian or mixed diet had almost similar levels of Cu. While the Zn level was slightly higher in women who adopted mixed diet as compared to those who adopted vegetarian diet. Also, women having habit of tobacco chewing had a lower level of serum Cu and a slightly higher level of Zn as compared to women with no such habits. In terms of caffeine consumption, it was observed that the caffeine consumers (in the form of tea or coffee) had significantly higher level of serum Cu and lower level of serum Zn compared to those who were not consuming caffeine (Table 3). Cu/Zn ratio was significantly higher in caffeine consumers.

Serum levels of Cu and Zn were correlated with the number of SAbs; it was observed that the serum level of Cu was significantly negatively correlated (r=-0.175, P=0.003) with the number of SAbs. As the number of SAb increased, the level of serum Cu decreased. No such correlation was observed between serum Zn and the number of SAbs (Table 4). It was also found that the number of SAb was significantly negatively correlated with Cu/Zn.

**Table 3:** Serum levels of Zn and Cu and their correlation with lifestyle habits


Variable	Serum Cu (mg/L)	Serum Zn (mg/L)	Cu/Zn

Vegetarian diet n=170	1.690 ± 0.05	1.403 ± 0.03	1.41 ± 0.070
Mixed diet n=107	1.672 ± 0.06	1.509 ± 0.04	1.28 ± 0.088
P value	0.777	0.091	0.250
Chewing habit n=15	1.591 ± 0.10	1.477 ± 0.10	1.12 ± 0.15
No chewing habit n=262	1.693 ± 0.04	1.442 ± 0.03	1.37 ± 0.057
P value	0.591	0.792	0.145
Caffeine consumption n=237	1.730 ± 0.04	1.471 ± 0.03	1.41 ± 0.060
No caffeine consumption n=40	1.439 ± 0.08^*^	1.641 ± 0.09^**^	1.06 ± 0.117^*^
P value	0.017	0.008	0.010


The data are presented as mean ± SE. *; P<0.05, **; P<0.01 show significant differences between two variables groups based on independent student’s t test, Cu; Copper, and Zn; Zinc.

**Table 4:** Correlation between trace metals levels and the number of SAb


Variable		Cu	Zn	Cu/Zn

Number of SAb				
	Pearson correlation	-0.175^**^	-0.006	-0.120^*^
	Sig. (2-tailed)	0.003	0.915	0.046
Cu	
	Pearson correlation		-0.040	0.698^**^
Sig. (2-tailed)		0.511	0.000
Zn				
	Pearson correlation			-0.601^**^
Sig. (2-tailed)			0.000


**; Correlation is significant at the 0.01 level (2-tailed), *; Correlation is significant at the 0.05 level (2-tailed), SAb; Spontaneous abortion, Cu; Copper, Zn; Zinc, and Sig.; Significance.

**Fig 1:**
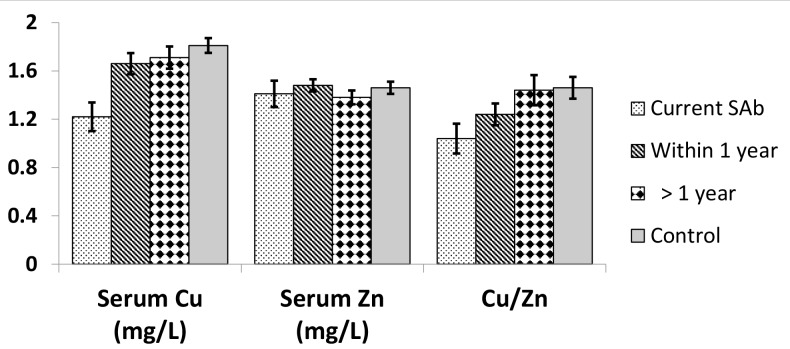
Level and ratios of trace metals (Cu and Zn) with respect to duration of last SAb faced. ***; P<0.001 shows significant differences compared to control based on independent student’s t test, Cu; Copper, Zn; Zinc, and SAb; Spontaneous abortion.

The data of trace metals was also analyzed with respect to the duration of the last SAb. The level of Cu was least significantly lower in women who had a recent SAb but highest in controls. No such pattern was observed in Zn serum level. It was also observed that Cu/Zn ratio was increasing as the duration of the last SAb decreased. Controls had the highest Cu/Zn ratio (Fig .1).

## Discussion

It is well known that both Cu and Zn are indispensable trace metals that are involved in important physiological functions of the human body. Their deficiencies lead to various diseases and disorders. However, excess levels of these elements can also lead to various pathological conditions. In the present study, both Cu and Zn level were lower in SAb group as compared to control, but differences were significant only in terms of Cu level. There are several reports which indicated that Zn has positive role in female reproduction as well as pregnancy outcomes (13-16). Further, Ajayi et al. (9) also found substantial decline in serum levels of Cu, Zn and vitamin E while a noteworthy elevation in serum levels of lead, selenium and cadmium in recurrent spontaneous abortion (RSA) cases compared to controls. Earlier, Jameson also reported that women who delivered pre-term (37^th^ week or earlier) or post-term (43^rd^ week or later), exhibited significantly lower serum levels of Zn during early pregnancy compared to women delivered a full-term (40^th^ week) pregnancy. Mothers with normal deliveries with normal infants exhibited significantly higher serum Zn but significantly lower serum Cu during early pregnancy compared to women with abnormal labors and immature infants (17). Later, Kiilholma et al. (18) reported that maternal serum Zn and calcium were lower in preterm subjects than in full-term groups and the cord Cu concentration and ceruloplasmin and their fetal/maternal ratios were significantly lower in women with preterm premature rupture of membranes (PPROM) compared to other groups. This indicates a role for Cu in PPROM and Zn in initiation of preterm labor, while calcium and iron may not be associated in the causation of prematurity or PPROM. Very recently, it was also found that Zn levels were lower in the sera of mothers with preterm deliveries with PPROM compared to those without PPROM; but Cu level did not differ between the groups either for maternal or umbilical cord serum or placental tissue (19). The mean serum levels of Cu and Zn were higher in pregnant women as compared to non-pregnant women at the time of subjects’ enrollment (cases-history of SAb and control subjects with children). Higher levels of Zn in pregnant women might be due to the presence of 73 non-pregnant subjects with a history of SAb out of a total 107 subjects in this group. Earlier, Izquierdo Alvares et al. (20) reported that serum Zn and Se levels decreased as gestation progresses, while serum Cu concentrations increased, and all the variation occurred mostly in the first 3 or 4 months.

Further, Zare et al. (21) reported that Zn deficiency may be one of the significant causes of adverse outcomes for lymphocyte immunotherapy (LIT) in RSA patients. Hence, compensation for Zn deficiency before LIT can be a promising approach to improve the immune response in patients with RSA. Earlier, Buamah et al. (22) found lower levels of maternal Cu in the abnormal pregnancies compared to the normal ones and these levels did not differ with increasing gestational age. Cu deficiency during pregnancy and post-natal development may have adverse effects on pregnancy and the developing fetus (23-26).

We found that Cu/Zn ratio was lower in SAb subjects than controls. Malavolta et al. (27) reported that the concentrations of Cu and Zn in serum are strictly regulated. There are several mechanisms that are responsible to decline serum concentration of Zn and raise serum concentration of Cu under inflammatory conditions. Recently, Shen et al. (11) observed statistically significant lower serum levels of Zn and iron in subjects with preterm delivery or miscarriage compared to control. Serum Zn levels were significantly lower in subjects with premature rupture of membrane while serum Cu, Zn, calcium and iron were significantly lower in subjects with intrauterine growth restriction.

## Conclusion

The present study together with the available information on this issue, suggests that Cu and Zn deficiency as well as Cu/Zn ratio might be associated with occurrence of SAb. Thus, further studies should be warranted on supplementation of these trace elements in clinical practices for the well being of pregnancy and pregnancy outcome.
